# COVID-19 Testing Equity in New York City During the First 2 Years of the Pandemic: Demographic Analysis of Free Testing Data

**DOI:** 10.2196/52972

**Published:** 2025-03-13

**Authors:** Daniel Rosenfeld, Sean Brennan, Andrew Wallach, Theodore Long, Chris Keeley, Sarah Joseph Kurien

**Affiliations:** 1 NYC Test & Treat Corps, The New York City Health + Hospitals Corporation, 50 Water St., New York, NY, 10004, United States, 1 212-788-3339, 1 212-788-3673; 2 Department of Housing Stabilization, The Massachusetts Executive Office of Housing and Livable Communities, Boston, MA, United States; 3 Memorial Sloan Kettering Cancer Center, New York, NY, United States; 4 Division of General Internal Medicine and Clinical Innovation, New York University Grossman School of Medicine, New York, NY, United States; 5 Department of Population Health, New York University, New York, NY, United States

**Keywords:** COVID-19 testing, health disparities, equity in testing, New York City, socioeconomic factors, testing accessibility, health care inequalities, demographic analysis, COVID-19 mortality, coronavirus, SARS-CoV-2, pandemic, equitable testing, cost, poor neighborhood, resources

## Abstract

**Background:**

COVID-19 has caused over 46,000 deaths in New York City, with a disproportional impact on certain communities. As part of the COVID-19 response, the city has directly administered over 6 million COVID-19 tests (in addition to millions of indirectly administered tests not covered in this analysis) at no cost to individuals, resulting in nearly half a million positive results. Given that the prevalence of testing, throughout the pandemic, has tended to be higher in more affluent areas, these tests were targeted to areas with fewer resources.

**Objective:**

This study aimed to evaluate the impact of New York City’s COVID-19 testing program; specifically, we aimed to review its ability to provide equitable testing in economically, geographically, and demographically diverse populations. Of note, in addition to the brick-and-mortar testing sites evaluated herein, this program conducted 2.1 million tests through mobile units to further address testing inequity.

**Methods:**

Testing data were collected from the in-house Microsoft SQL Server Management Studio 18 Clarity database, representing 6,347,533 total tests and 449,721 positive test results. These tests were conducted at 48 hospital system locations. Per capita testing rates by zip code tabulation area (ZCTA) and COVID-19 positivity rates by ZCTA were used as dependent variables in separate regressions. Median income, median age, the percentage of English-speaking individuals, and the percentage of people of color were used as independent demographic variables to analyze testing patterns across several intersecting identities. Negative binomial regressions were run in a Jupyter Notebook using Python.

**Results:**

Per capita testing inversely correlated with median income geographically. The overall pseudo *r*^2^ value was 0.1101 when comparing hospital system tests by ZCTA against the selected variables. The number of tests significantly increased as median income fell (SE 1.00000155; *P<*.001). No other variables correlated at a significant level with the number of tests (all *P* values were >.05). When considering positive test results by ZCTA, the number of positive test results also significantly increased as median income fell (SE 1.57e^–6^; *P*<.001) and as the percentage of female residents fell (SE 0.957; *P*=.001). The number of positive test results by ZCTA rose at a significant level alongside the percentage of English-only speakers (SE 0.271; *P*=.03).

**Conclusions:**

New York City’s COVID-19 testing program was able to improve equity through the provision of no-cost testing, which focused on areas of the city that were disproportionately impacted by COVID-19 and had fewer resources. By detecting higher numbers of positive test results in resource-poor neighborhoods, New York City was able to deploy additional resources, such as those for contact tracing and isolation and quarantine support (eg, free food delivery and free hotel stays), early during the COVID-19 pandemic. Equitable deployment of testing is feasible and should be considered early in future epidemics or pandemics.

## Introduction

In March 2020, New York City (NYC) emerged as one of the global epicenters of SARS-CoV-2 infections and COVID-19 deaths. This was roughly 2 months after the first detected case in the United States and 3 months following the first case in Wuhan, China [1]. Early in the pandemic, within the United States and especially within NYC, testing availability was extremely limited. In fact, there were no positive test results recorded in NYC until the beginning of March 2020 [2]. Over the ensuing 3 years, over 45,000 deaths and over 3.2 million infections were documented in NYC—a wave of mortality that contributed to a decline of 4.6 years in life expectancy from 2019 to 2020 alone [3].

It should be noted that COVID-19 mortality and SARS-CoV-2 infection rates have varied widely based on several spatial and socioeconomic factors [4]. US counties with higher poverty rates had higher COVID-19 case numbers and related death rates, and racial and ethnic minority individuals are at increased vulnerability for COVID-19 when considering both infection rates and mortality rates [5,6]. While life expectancy declined by 4.6 years citywide, it declined by 6 years for Hispanic and Latinx New Yorkers (to 77.3 years), by 5.5 years for Black New Yorkers (to 73 years), and by 3 years for White New Yorkers (to 80.1 years) [3]. These mortality disparities were not limited to race. Individuals with chronic conditions, such as obesity and diabetes, faced higher mortality rates than those faced by the general population as a whole, while socioeconomic factors, such as poverty, housing overcrowding, the effects of historical residential racial segregation, and an inability to perform some jobs remotely, have intersected with the aforementioned chronic health issues to create a perfect storm of increased COVID-19 mortality in particular neighborhoods [7].

Following the initial lack of testing at the onset of the pandemic, the federal and state governments sought to develop and implement a mass testing strategy. Once tests were made available, significant barriers to accessing testing remained in terms of costs and the geographic locations of testing sites. The NYC Health + Hospitals Corporation (H+H)—the largest public health system in the United States, with 11 acute care facilities, 6 diagnostic and treatment centers, and more than 50 neighborhood health centers (mostly, but not entirely, in economically depressed neighborhoods)—became the largest free testing provider in NYC. The NYC Department of Health and Mental Hygiene has always tracked health and socioeconomic disparities. This background provided the basis for establishing the TRIE (Taskforce on Racial Inclusion & Equity) neighborhoods [8]. These were neighborhoods that had economic disadvantages and were disproportionately impacted by COVID-19. We leveraged the neighborhoods, which essentially involved combining former disparity data related to the impact of COVID-19, to help set our strategy for allocating testing sites. By providing access to no-cost COVID-19 testing, we sought to understand whether our equitably distributed testing program had a relationship with health outcomes and whether the no-cost testing option can be used to increase testing in underserved areas.

## Methods

### Data Sources

Data on COVID-19 testing that was directly administered by the NYC H+H were collected from the in-house NYC H+H Microsoft SQL Server Management Studio 18 Clarity database, representing 6,347,533 total tests and 449,721 positive test results. These tests were sourced from the in-house NYC H+H instance of Epic Systems; these did not include tests conducted by external testing vendors contracted by the NYC H+H or tests conducted by the school testing program that mandated surveillance testing in NYC public schools from 2020 to 2022. Demographic data were taken from 2020 US Census Bureau data [9]. Citywide testing data came from the NYC coronavirus data repository [10].

### Experimental Design

We initially sought to run a Poisson regression analysis comparing counts of tests and positive test results to demographic variables while using population as an offset; however, we found the data to be overdispersed and chose to use negative binomial regressions. In terms of the variables we chose to analyze, we were influenced by two studies from King County, Washington, and NYC that, among other methods, analyzed testing rates against demographic variables. The King County, Washington, study used a variable called “people of color (POC),” into which POC racial data were collapsed due to collinearity among POC groups [11]. Given similar patterns of collinearity in our data, we chose to do this in our study. The NYC study used a number of different demographic variables, such as income, education, and housing overcrowding, to calculate a single socioeconomic index score [2]. Although this is one way to handle collinearity between socioeconomic variables, such as income and poverty, we chose to use median income as our main predictor of socioeconomic status. As variables related to COVID-19 mortality and testing rates, we included age and sex breakdowns, as being older and being male positively correlate with higher mortality. As a measure of presumed difficulty in accessing health care, the percentage of English speakers was included.

### Ethical Considerations

We used deidentified patient data. The Biomedical Research Alliance of New York (BRANY) Institutional Review Board (IRB) determined that our research in this paper did not constitute research involving human subjects that is regulated by US Department of Health and Human Services or US Food and Drug Administration regulations, and it was therefore not subject to further BRANY IRB review. The BRANY study ID is 23-15-564, the sponsor ID is RH2023-814, and our category was determined as “15-Not Human Subject Research.” We were advised by BRANY that we did not have to create a National Clinical Trial number with the US National Library of Medicine ClinicalTrials.gov website because our data-only study used preexisting data.

### Analysis

Regressions were run in a Jupyter Notebook (Project Jupyter) using Python. Data visualizations were created by using Tableau (Tableau Software LLC). A selection of data visualizations can be found in the *Results* section. As this was a spatial analysis, we took care to map each of our demographic variables, testing data points, and background health outcome data. We mapped out “Percent POC,” “Percent Female,” “Percent Speaks Only English,” “Median Age,” “Median Income,” and “COVID-19 death rate” as demographic and health outcome variables by zip code tabulation area (ZCTA). To spatially display our testing data, we mapped the variables “Total Citywide Tests per 100,000,” “NYC H+H COVID-19 Tests,” “NYC H+H COVID-19 tests per 100,000,” “NYC H+H positive tests per 100,000,” and “NYC H+H COVID-19 test positivity rate” also by ZCTA. We also created scatter plots to illustrate the relationships between these variables.

## Results

**Figure 1.**
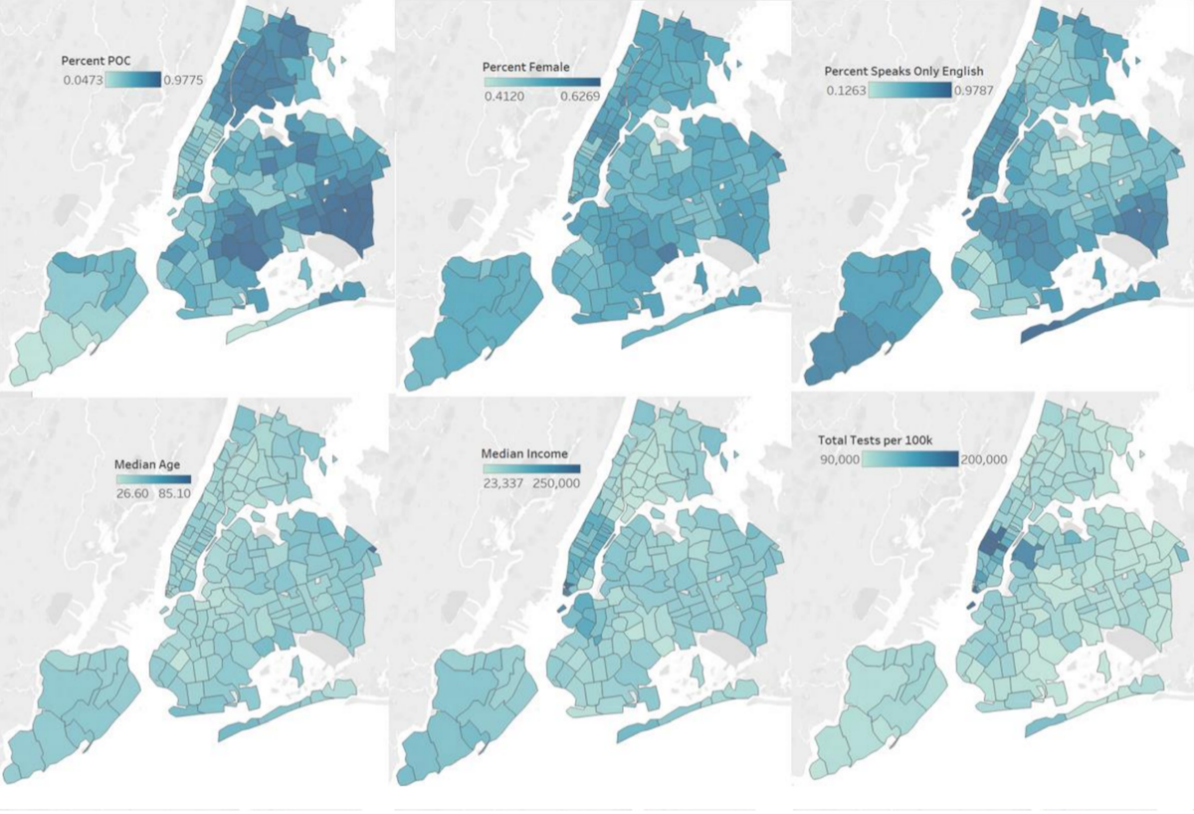
Selected demographic, testing, and mortality variables by zip code tabulation areas (clockwise from upper left): “Percent POC,” “Percent Female,” “Percent Speaks Only English,” “Total Tests per 100k,” “Median Income” (US $), and “Median Age” (years). POC: people of color.

**Figure 2.**
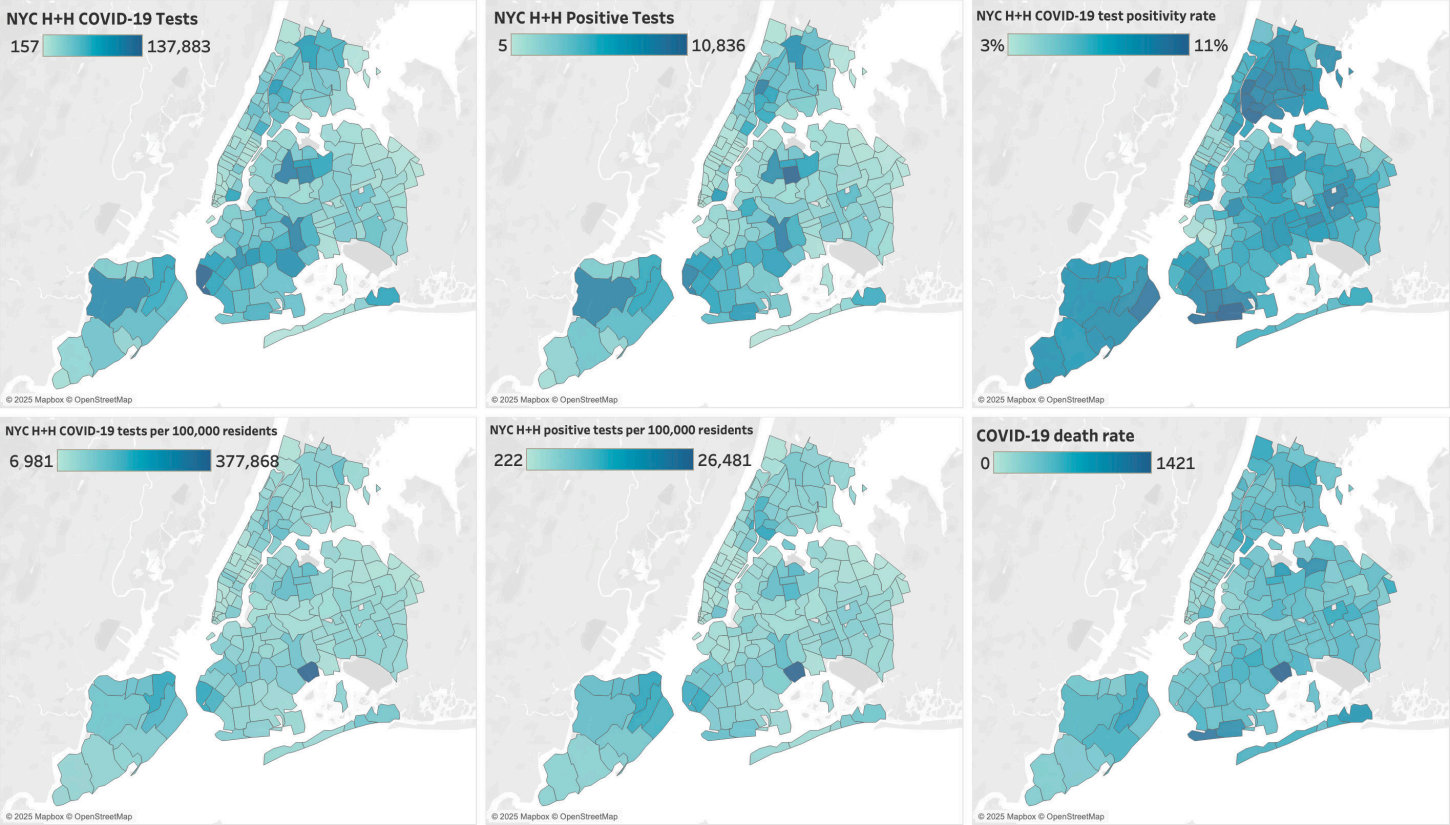
Selected demographic, testing, and mortality variables by zip code tabulation areas continued (clockwise from upper left): “NYC H+H COVID-19 Tests,” “NYC H+H Positive Tests,” “NYC H+H COVID-19 test positivity rate,” “COVID-19 death rate,” “NYC H+H positive tests per 100,000,” and “NYC H+H COVID-19 tests per 100,000.” NYC H+H: New York City Health + Hospitals Corporation.

**Figure 3.**
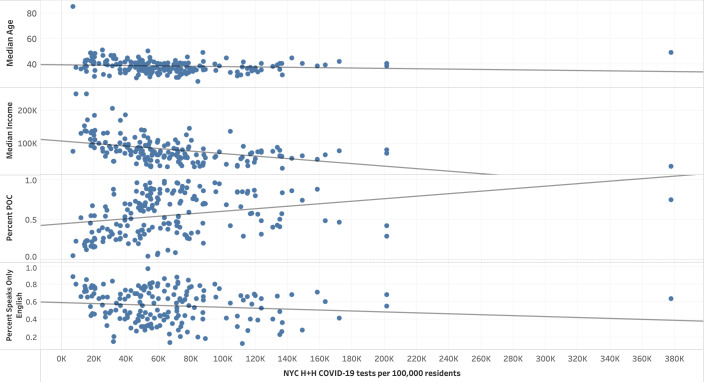
Selected demographics compared to NYC H+H COVID-19 tests per 100,000 residents by zip code tabulation area (from top to bottom): “Median Age” (years), “Median Income” (US $), “Percent POC,” and “Percent Speaks Only English” by NYC H+H COVID-19 tests per 100,000 residents. NYC H+H: New York City Health + Hospitals Corporation; POC: people of color.

**Figure 4.**
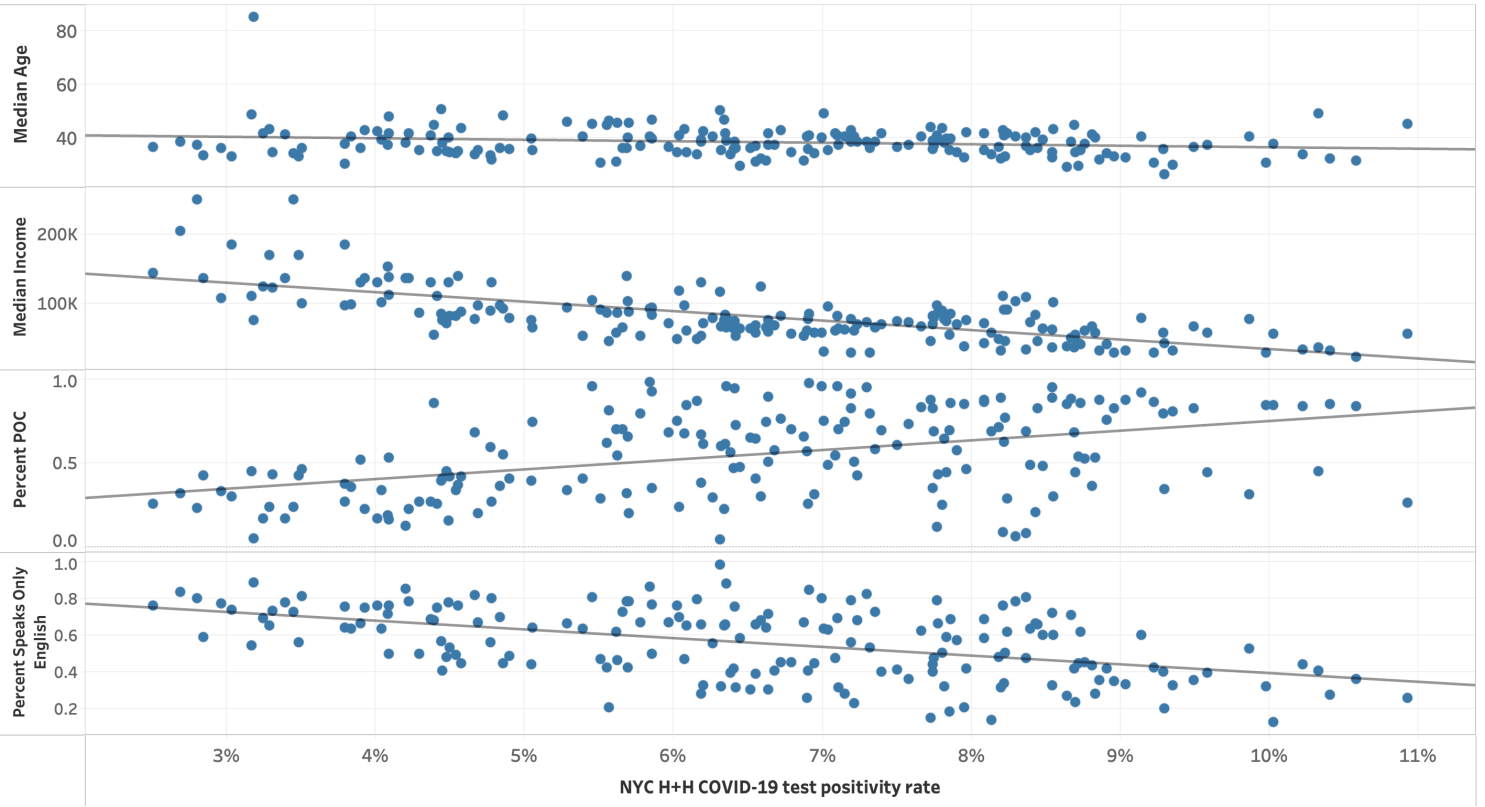
Selected demographics compared to NYC H+H COVID-19 test positivity rate by zip code tabulation area (from top to bottom): “Median Age” (years), “Median Income” (US $), “Percent POC,” and “Percent Speaks Only English” by NYC H+H test positivity rate. NYC H+H: New York City Health + Hospitals Corporation; POC: people of color.

**Figure 5.**
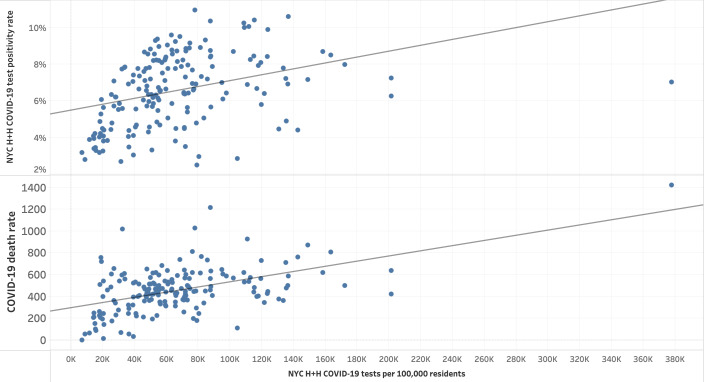
Selected test and death rates compared to NYC H+H COVID-19 tests per 100,000 residents by zip code tabulation area (from top to bottom): NYC H+H COVID-19 test positivity rate and COVID-19 death rate by NYC H+H COVID-19 tests per 100,000 residents. NYC H+H: New York City Health + Hospitals Corporation.

**Figure 6.**
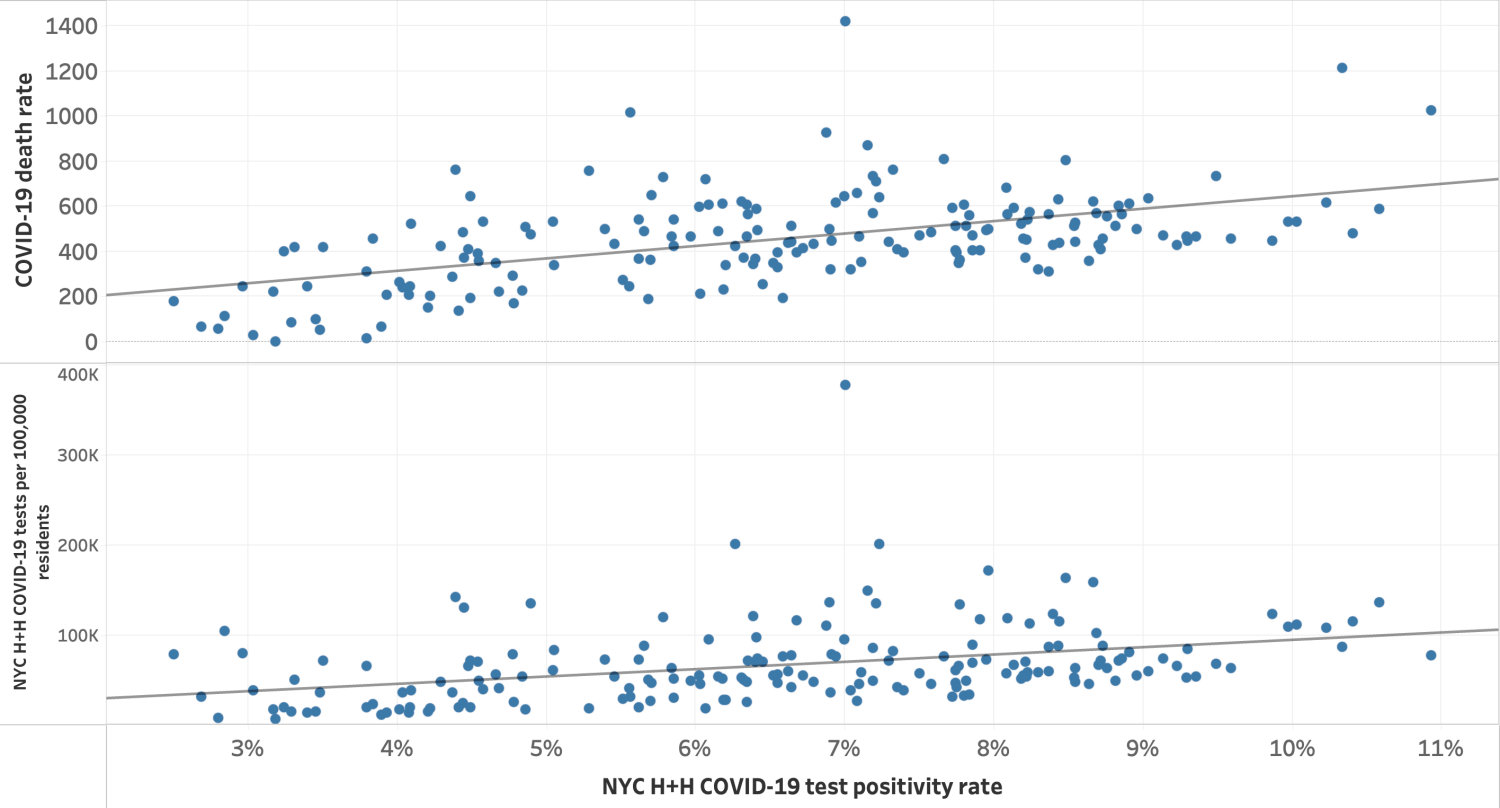
Selected test and death rates compared to NYC H+H COVID-19 positivity rate by zip code tabulation area (from top to bottom): COVID-19 death rate and NYC H+H COVID-19 tests per 100,000 residents by NYC H+H COVID-19 positivity rate. NYC H+H: New York City Health + Hospitals Corporation.

The overall pseudo *r*^2^ value was 0.1101 when comparing NYC H+H tests by ZCTA against the selected variables. The number of NYC H+H tests significantly increased as median income fell (exponentiated β=.99998, SE 1.00000155; *P*<.001). No other variables correlated at a significant level with the number of tests (all *P* values were >.05). When considering positive test results by ZCTA, the number of positive test results also significantly increased as median income fell (exponentiated β=.99998, SE 1.57e^–6^; *P*<.001) and as the percentage of female residents fell (exponentiated β=.04014, SE 0.957; *P*=.001). Positive test results by ZCTA rose at a less significant level alongside the percentage of English-only speakers (β=1.81630, SE 0.271; *P*=.03). No other predictor variables correlated with positive test results by ZCTA (all *P* values were >.05). *P*<.05 was chosen as a standard biomedical level of significance for geographic area–level research. A selection of data visualizations depicting our results are shown in Figures 1-6.

## Discussion

The devastatingly high mortality rates seen in NYC during the height of the first COVID-19 wave in April 2020 were likely linked to undetected cases as a result of limited testing capabilities at the time. Roughly half of all deceased NYC patients with COVID-19 died during the first months of the first wave, making comparative analysis somewhat difficult in NYC when compared to other regions. Approximately 6 million COVID-19 tests were completed by the NYC H+H between early 2020 and late 2022, though most testing was done following the first wave in 2020 due to the time and resources required to set up testing sites. In our analysis, the number of NYC H+H no-cost COVID-19 tests increased as the median income of a discrete neighborhood fell—a trend that stood in opposition to overall citywide testing trends, wherein the highest testing rates were observed in wealthier areas of the city. The number of positive test results at the NYC H+H also rose as median income fell in a discrete neighborhood, even when controlling for other variables. This suggests that NYC H+H no-cost COVID-19 tests were equitably distributed in targeted increased-risk neighborhoods of NYC. This was important, considering the disproportionate negative health impacts of the pandemic on these neighborhoods, which already had a preexisting vulnerability to a range of adverse health outcomes.

Consistent and easily accessible large-scale testing may be a crucial element of successful strategies for avoiding additional mortality. Our research echoes that of some other studies that found an inverse relationship between COVID-19 positivity and test availability [11]. With regard to the racial and income equity issues that have been seen in COVID-19 testing within NYC and the United States, our findings on tests administered by the NYC H+H were more mixed for demographic variables, such as “Percent POC,” that otherwise might be related to COVID-19 mortality risk. Our study had limitations with regard to interpreting disparities in COVID-19 testing rates across space and disparities in time to COVID-19 mortality. COVID-19 testing was extremely limited early in the pandemic due to the lack of readily available testing, whereas from 2022 to 2023, at-home rapid testing became ubiquitous but came with the caveat that results were rarely reported. In addition, as we compared COVID-19 testing rates and results between geographic and demographic variables, we recognized the pitfalls that may arise when equating jurisdiction-level data with individuals who live within those jurisdictions. NYC is an extremely diverse city with substantial income inequality and variability even within the smallest geographic neighborhoods. The relationship between COVID-19 test positivity and the percentage of female residents could suggest that ZCTAs with a higher percentage of male residents had higher positivity rates, and this information could be used to inform future testing campaign strategies (eg, targeting areas with a higher percentage of male residents).

We take the inverse relationship between NYC H+H testing rates and median income as a positive sign that the no-cost COVID-19 testing program was able to penetrate areas of the city that were likely neglected by other testing providers. Despite the devastating consequences from the COVID-19 pandemic in NYC, this targeted, equitably distributed testing plan could have broader public health implications if similar outreach strategies are used in future pandemics.

## Supplementary material

10.2196/52972 Multimedia Appendix 1 Python code.

## Abbreviations

BRANY: Biomedical Research Alliance of New York
H+H: Health + Hospitals Corporation
IRB: Institutional Review Board
NYC: New York City
POC: people of color
TRIE: Taskforce on Racial Inclusion & Equity
ZCTA: zip code tabulation area
