# Supplementary material for: COVID-19 Testing Equity in New York City During the First 2 Years of the Pandemic: Demographic Analysis of Free Testing Data
Source: JMIR Public Health Surveill. 2025 Mar 13;11:e52972. doi: 10.2196/52972 (PMC11924968; doi:10.2196/52972)
Supplement: Multimedia Appendix 1 [file publichealth-v11-e52972-s001.docx]

## Multimedia Appendix

All underlying data by ZCTA can be found on GitHub at

https://github.com/rosenfed/testingequitydata/blob/main/2022-12-02%20updated%20testing%20equity%20python%20code.ipynb

import pandas as pd

import numpy as np

import statsmodels.api as sm

from statsmodels.genmod.families import Poisson

import matplotlib.pyplot as plt

import scipy.stats as stats

from sklearn.linear_model import PoissonRegressor

plt.rcParams['figure.figsize'] = (20.0, 10.0)

acsimport = pd.read_csv (r"ACSimport4.csv")

#negative binomial model, total tests

famnegbin = sm.families.NegativeBinomial()

offset = np.log(acsimport['total_pop'])

exog = acsimport[['Median Income', 'Median Age', 'Percent Female', 'Percent POC', 'Speaks only

English %']]

endog = acsimport[['updatedhhctests']]

glm_negbin = sm.GLM(endog,

exog,M=sm.robust.norms.HuberT(),family=famnegbin,link='log',offset=offset)

negbin_results=glm_negbin.fit(cov_type="hc1")

print(negbin_results.summary())

# exponentiating results for total tests, repeat for positive tests

print('Median Income')

print(np.exp(-1.007e-05))

#print(np.exp(-0.000),np.exp(0.000))

print('Median Age')

print(np.exp(-0.0115))

#print(np.exp(-0.060),np.exp(-0.048))

print('Percent Female')

print(np.exp(1.0481))

#print(np.exp(-0.000),np.exp(0.000))

print('Percent POC')

print(np.exp(-0.0228 ))

#print(np.exp(-0.0000966),np.exp(-0.000))

print('Speaks only English %')

print(np.exp( 0.4689))

#print(np.exp(-0.000),np.exp(0.000))

#negative binomial model positive casesfamnegbin = sm.families.NegativeBinomial()

offset = np.log(acsimport['total_pop'])

exog = acsimport[['Median Income', 'Median Age', 'Percent Female', 'Percent POC', 'Speaks only

English %']]

endog = acsimport[['updatedhhcpositivetests']]

glm_negbin = sm.GLM(endog,

exog,M=sm.robust.norms.HuberT(),family=famnegbin,link='log',offset=offset)

negbin_results=glm_negbin.fit(cov_type="hc1")

print(negbin_results.summary())
